# Protective Epitopes of the *Plasmodium falciparum* SERA5 Malaria Vaccine Reside in Intrinsically Unstructured N-Terminal Repetitive Sequences

**DOI:** 10.1371/journal.pone.0098460

**Published:** 2014-06-02

**Authors:** Masanori Yagi, Gilles Bang, Takahiro Tougan, Nirianne M. Q. Palacpac, Nobuko Arisue, Taiki Aoshi, Yoshitsugu Matsumoto, Ken J. Ishii, Thomas G. Egwang, Pierre Druilhe, Toshihiro Horii

**Affiliations:** 1 Department of Molecular Protozoology, Research Institute for Microbial Diseases, Osaka University, Suita, Osaka, Japan; 2 Laboratoire de Parasitologie Bio-Médicale, Institut Pasteur, Paris, France; 3 Laboratory of Adjuvant Innovation, National Institute of Biomedical Innovation, Ibaraki, Osaka, Japan; 4 Laboratory of Vaccine Science, Immunology Frontier Research Center, Osaka University, Suita, Osaka, Japan; 5 Laboratory of Molecular Immunology, School of Agriculture and Life Sciences, The University of Tokyo, Tokyo, Japan; 6 Med Biotech Laboratories, Kampala, Uganda; Université Pierre et Marie Curie, France

## Abstract

The malaria vaccine candidate antigen, SE36, is based on the N-terminal 47 kDa domain of *Plasmodium falciparum* serine repeat antigen 5 (SERA5). In epidemiological studies, we have previously shown the inhibitory effects of SE36 specific antibodies on *in vitro* parasite growth and the negative correlation between antibody level and malaria symptoms. A phase 1 b trial of the BK-SE36 vaccine in Uganda elicited 72% protective efficacy against symptomatic malaria in children aged 6–20 years during the follow-up period 130–365 days post–second vaccination. Here, we performed epitope mapping with synthetic peptides covering the whole sequence of SE36 to identify and map dominant epitopes in Ugandan adult serum presumed to have clinical immunity to *P. falciparum* malaria. High titer sera from the Ugandan adults predominantly reacted with peptides corresponding to two successive N-terminal regions of SERA5 containing octamer repeats and serine rich sequences, regions of SERA5 that were previously reported to have limited polymorphism. Affinity purified antibodies specifically recognizing the octamer repeats and serine rich sequences exhibited a high antibody-dependent cellular inhibition (ADCI) activity that inhibited parasite growth. Furthermore, protein structure predictions and structural analysis of SE36 using spectroscopic methods indicated that N-terminal regions possessing inhibitory epitopes are intrinsically unstructured. Collectively, these results suggest that strict tertiary structure of SE36 epitopes is not required to elicit protective antibodies in naturally immune Ugandan adults.

## Introduction

Despite the vast malaria burden no effective malaria vaccine exists [1], [2]. The development of malaria vaccines has mainly focused on *Plasmodium falciparum*, the most deadly of five *Plasmodium* species that infect humans. Malaria vaccine development strategies vary depending on the target stages of the parasite life cycle, i.e. sporozoite, intra-hepatocytic stage, asexual erythrocyte stages, gametocyte, and mosquito midgut stages. Asexual erythrocyte stage antigens are thought to elicit antibodies which reduce blood parasitemia and lessen the severity of malaria symptoms. However, sequence polymorphism of many antigens, as observed in several vaccine candidates such as merozoite surface protein (MSP)-1, MSP-2 [3] and apical membrane antigen-1 (AMA-1) [4], hamper the systematic vaccine development strategy based on host immune responses against malaria parasites.

*P. falciparum* serine repeat antigen 5 (*Pf*SERA5) is one of the candidate vaccines in human trial [5]–[7]. Abundantly expressed in the parasitophorous vacuole and on the merozoite surface, and belonging to the SERA protein family, the 120 kDa protein is processed into 47, 50, 6 and 18 kDa domains at the time of schizont rupture. While the 50 and 6 kDa domains are secreted, the 47 and 18 kDa domains are covalently linked by disulfide bond(s) and remain on the merozoite surface [7]–[9]. *Pf*SERA5 was the first physiological substrate identified for *P. falciparum* subtilisin-like serine protease (*Pf*SUB1) [10].

Sequence analysis of 445 *P. falciparum* field isolates from nine countries worldwide revealed that sequence polymorphism of *Pf*SERA5 is remarkably limited unlike other malaria vaccine candidates [11]. Moreover, high antibody level against the N-terminal 47 kDa domain correlated with the absence of fever or low parasitemia [5], [12], [13]. Under *in vitro* conditions, antibodies against the N-terminal domain were also suggested to correlate with antiparasitic effects through several mechanisms. At high antibody concentration, inhibition of parasite growth was found to be associated with merozoite agglutination [14] or complement mediated cell lysis of segmented schizont [15]. At low antibody concentrations, monocyte-mediated antibody dependent cellular inhibition (ADCI) activity has been demonstrated [16].

SE36 is based on the N-terminal 47 kDa domain constructed by removing the polyserine region located in the middle of the domain [5]. A recent phase 1 b clinical trial and follow-up study 365 days post-second vaccination elicited 72% protective efficacy against symptomatic malaria in Ugandan children aged 6–20 years [6]. Although the exact function of *Pf*SERA5 remains unknown, a parasite inhibitory epitope defined by a murine monoclonal antibody was mapped onto amino acids 17–73 of the Honduras-1 strain, a well conserved region in diverse geographical isolates of *P. falciparum* [17], [18].

Intrinsically unstructured proteins (IUPs), also called intrinsically disordered proteins or natively unfolded proteins, have for the past 10–20 years generated interest because of their unusual way to carry out molecular recognition different from traditional protein structure-function paradigms. IUPs contain polypeptide chains lacking stable tertiary structure when they exist alone, however, some of them are known to switch to more ordered conformation upon recognition of their binding partners and play their biological roles [19], [20].

*Pf*SERA5 (17–73) has an octamer repeat (OR) region and a serine rich (SR) region. The repeat number of octamer motifs varies depending on strains but the basic motif sequences are well conserved in *P. falciparum* [11]. These OR and SR regions have biased amino acid composition and are low complexity regions with little diversity in their amino acids [21]. Low complexity regions are often found in *Plasmodium* species and, due to lack of hydrophobic amino acids, such regions are expected to be intrinsically unstructured [22]. *P. falciparum* proteins, such as MSP-2 and trophozoite exported protein 1 (Tex1), are reported to have intrinsically unstructured regions (IURs) [23]–[25]. P27A, an IUR found in Tex-1, was well recognized by sera from individuals living in malaria endemic areas. Moreover, murine antibodies purified from P27A immunized mice showed high ADCI activities [24]. Immunogenicity of IUR in naturally infected humans was also reported for *P. vivax* AMA-1 [26].

In the present study, we performed epitope mapping with overlapping synthetic peptides covering the whole sequence of SE36 utilizing serum from Ugandan volunteers, and serum from previously vaccinated mice and squirrel monkeys. We identified the N-terminal repetitive sequence regions of SE36 as immunodominant IgG epitopes in Ugandan individuals presumed to have clinical immunity to *P. falciparum* malaria. We have demonstrated previously that antibodies raised against N-terminal region of *Pf*SERA5 strongly inhibit *in vitro* parasite growth by ADCI at concentrations which do not show any detectable direct inhibition of growth [16], thus we used this assay as a screen for functional inhibition activity of anti-SE36 IgG. Affinity purified antibodies against N-terminal repetitive sequence regions of SE36 showed high ADCI activities suggesting that the regions are protective epitopes. Additionally, the OR and SR regions are revealed to be intrinsically unstructured by spectroscopic methods and protein structure predictions. These results show that the N-terminal repetitive sequences have characteristics of an intrinsically unstructured region and are highly immunogenic in Ugandan adults eliciting protective antibodies against malaria.

## Materials and Methods

### Ethics Statement

Serum samples from pool of individuals living in endemic areas and individual Ugandan serum samples were from participants in an earlier epidemiological study [9]. Briefly, the study utilizes residual samples from a cross-sectional study of 40 (37 sera are available for this study) healthy Ugandan adults living in Atopi Parish, a malaria holoendemic area, located 5 km west of Apac Town, 300 km north of Kampala. Ethical clearance for sampling and consent was obtained and approved by the Uganda National Council for Science and Technology under the 1997 Guidelines for Health Research Involving Human Subjects in Uganda [9]. In agreement with the local community leadership, a process of dialogue was done. Information about the study was given to the head of the community, household and study participants. Verbal consent was obtained for voluntary participation and for blood samples to be taken and stored for use in future studies. It was deemed culturally-sensitive in this community that experienced recent government conflict that verbal informed consent be sought (written consent were not practiced, disliked and viewed as mistrust). Being a cross-sectional study, signatures will also be the only record of their participation and risk of privacy is minimized if their signature is not recorded. No other records exist for their participation. Blood samples were coded during blood collection, processed within a few hours after collection and separated into sera, which was stored at −20°C and −70°C until analyses.

Animal housing, care and handling of squirrel monkeys were done in strict compliance with “The guidelines for the care and use of laboratory animals” by the University of Tokyo [5]. Briefly, male squirrel monkeys (*Saimiri sciureus*) of Guyana phenotype were bred in captivity. The monkeys were quarantined and conditioned for at least a month prior to the commencement of the study. Thorough medical examinations revealed that the animals were free of all intestinal and any blood stage infections including malaria, and they were declared to be in a general good health by a veterinarian. They were housed in the Amami Laboratory of Injurious Animals, Institute of Medical Science, University of Tokyo in individual safety cabinets with an exercise bar at a controlled environment of 24±2°C and 50±10% humidity. Monkeys were fed with new world monkey chow (Clea Japan Inc., Tokyo, Japan) and allowed free access to water. Lighting was automatically regulated on a 12 hours light-dark cycle. The monkeys, weighing between 680 and 760 g at the beginning of the experiment, were divided into two treatment groups that received their intramuscular injection on the left thigh 5 and 3 weeks before challenge infection. All procedures were performed under anesthesia and all efforts were made to minimize suffering. All experimental procedures were approved by the School of Agriculture and Life Sciences, the University of Tokyo. Additional details of animal welfare/care and steps taken to ameliorate suffering were in accordance with the recommendations of the Weatherall report, “The use of non-human primates in research”. During the study no monkey died or was sacrificed.

Animal experiments using mice were approved by the Animal Care and Use Committee of the Research Institute for Microbial Diseases, Osaka University, Japan. Mice care and steps to ameliorate suffering was conducted in accordance with the guidelines of the committee and immunization experiments were in accordance with the GERBU adjuvant protocol described below (GERBU Biotechnik GmbH, Heidelberg, Germany). During the study, no mice were sacrificed.

### Animal Blood Samples

Residual serum samples from squirrel monkeys (*Saimiri sciureus*) that received 50 µg SE36 protein with 500 µg aluminum hydroxide gel in 0.5 ml of PBS and those in the control group that received the same volume of PBS by intra-muscular injection were utilized [5]. In brief, after 2 or 3 immunizations, these monkeys were followed through after *P. falciparum* challenge infection [5]. For mouse immunization, 30 ddY mice were purchased from Japan SLC, Inc. (Hamamatsu, Japan). Each mouse was subcutaneously immunized with 50 µl of 1 mg/ml SE36 protein and 50 µl GERBU adjuvant (100 µl in total) 4 times at 2-week intervals. Two weeks after last immunization, blood draw was performed from the mouse tail and blood samples from the mice were pooled for the experiments.

### Recombinant SE36 Protein

GMP grade SE36 protein was expressed in *E. coli* using a codon optimized synthetic gene and purified as previously described [5].

### Synthetic Peptides

**Figure 1:**
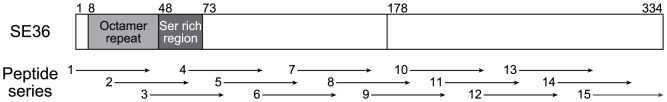
Schematic representation of synthetic peptide series I covering the whole sequence of SE36 protein. The number 178 denotes the position of polyserine sequence present in *Pf*SERA5 but deleted in SE36 [5].

Fifteen synthetic peptides of 40–42 residues covering the whole sequence of SE36 protein were synthesized by Operon Biotechnology Inc. (Tokyo, Japan) (Fig. 1 and Table S1, series I). Each peptide was designed to overlap with two adjacent peptides at its N- and C-terminal halves, respectively.

### Enzyme-Linked Immunosorbent Assay

Enzyme-linked immunosorbent assay (ELISA) was performed using flat-bottomed 96-well Nunc-Immuno plates (Nunc, Roskilde, Denmark). SE36 protein or the synthetic peptides were dissolved in carbonate buffer (pH 9.6) as coating buffer at a concentration of 0.1 µg/ml. For ELISA assays of synthetic peptides, each plate was coated with the whole peptide series. The plates were coated overnight at 4°C with 100 µl of the protein or peptide solutions, washed three times with PBS containing 0.05% Tween 20 (PBS/T) and blocked for an hour with 5% skim milk in PBS at 37°C. The plates were again washed three times with PBS/T prior to addition of serum samples or purified IgG prepared in 5% skim milk in PBS/T. Test samples were added to wells at optimized concentration and incubated for an hour at 37°C. After washing with PBS/T, peroxidase-conjugated goat IgG fraction to human IgG (whole molecule) (55220; Cappel ICN Pharmaceuticals Inc, Aurora, OH) diluted 1∶2000; or horseradish peroxidase-conjugated rabbit anti-human IgG antibody (A8792; Sigma-Aldrich Corp., St. Louis, MO) diluted 1∶2000; or peroxidase conjugated affiniPure goat anti-mouse IgG antibody (H+L) (115-035-166; Jackson ImmunoResearch Laboratories, Inc., West Grove, PA) diluted 1∶5000 in 5% skim milk in PBS/T was added to the plates and incubated at 37°C for 1 hour. The plates were washed and incubated with 100 µl freshly prepared citrate-phosphate buffer (pH 5.0) containing 0.2% hydrogen peroxide and OPD tablet (154-01673; Sigma-Aldrich Corp., St. Louis, MO) for 15 minutes. The reaction was stopped with 100 µl of 2 M sulfuric acid and optical density was read at 492 nm.

### Purification of Antibodies

To prepare anti-OR and anti-SR antibodies for ADCI experiments, the antibodies were purified from Ugandan high antibody titer serum pool (SE36-positive serum pool) [9]. Prior to the purification of antibodies specific to the OR or SR region, whole antibodies were purified from the serum pool with HiTrap Protein G HP columns (GE Healthcare UK Ltd, Buckinghamshire, UK). Antibodies purified by Protein G columns were then loaded to either OR or SR-specific peptide columns. The OR/SR peptide columns were prepared with SulfoLink Immobilization Kit for Peptides (Thermo Fisher Scientific, Waltham, MA), so that OR/SR peptides with cysteine residues at the N-termini (series II peptides 1 and 3, Table S2) were immobilized to the columns via thiol groups, following recommendations of the manufacturer. Antibodies bound to the columns were eluted with 0.1 M Gly-HCl at pH 2.7 and immediately neutralized with 1 M Tris-HCl at pH 8.5.

Murine serum pool from SE36-vaccinated mice was used to purify the antibody against the whole SE36 molecule. However, due to limited sample volume, after applying to Protein G column no further purification was done. All antibodies were dialyzed against RPMI 1640 (Nakalai Tesque, Kyoto, Japan) prior to ADCI assays.

### Antibody-Dependent Cellular Inhibition Assay (ADCI) and Assessment of Parasitemia by Flow Cytometry

The ADCI assay was as previously described [27], [28] and carried out with either (i) human IgG purified with protein G and then affinity purified for specific peptides; or (ii) protein G purified IgG from mice immunized with SE36. Final concentration of IgG was at 0.3 mg/ml. As a positive control, a pool of hyperimmune African adults IgG (PIAG) [27] was used at 2 mg/ml to assess reproducibility between each assay. Monocytes (MN) from peripheral blood mononuclear cells were further enriched using EasySep Human Monocyte Enrichment Kit Without CD16 Depletion according to manufacturer's instruction (StemCell Technologies Inc., Vancouver, BC, Canada). Monocyte monolayer was obtained after incubation of 2×10^5^ MN for 30 minutes at 37°C in 5% CO_2_ atmosphere. A synchronized asexual blood stage parasite culture (K1 clone) with very mature schizonts (0.5% parasitemia, 2.5% haematocrit) was added on the MN monolayer in addition to murine and human IgG to be tested. Intrinsic anti-parasitic effect of control and test IgG sera was assessed in wells containing the blood stage parasites without MN. Prior to ADCI assay, only MN with non-significant phagocytosis effect against *in vitro* growth of asexual blood stage parasites were selected. Samples were tested in duplicate wells. Plates were incubated in a candle jar at 37°C, in a 5% CO_2_ incubator. At 48 and 72 hours, 50 µl of complete medium was added to each well. At 96 hours the assay was stopped and the parasitemia determined by flow cytometry (FACSCalibur, BD Biosciences, CA). Assays were performed at the same day by two independent researchers.

Flow cytometry enumeration of infected erythrocytes with viable malaria parasites was performed by double staining of DNA and RNA using hydroethidine (HE) and thiazole orange (TO) (Sigma-Aldrich Corp.). Briefly, erythrocytes were incubated for 20 min at 37°C in the dark with 20 µg/ml of HE diluted in PBS-1% FCS (FACS buffer), washed three times in FACS buffer, followed by another incubation for 30 min at room temperature in the dark with TO diluted at 1∶15000 in FACS buffer. Analysis was performed on 1×10^5^ erythrocytes with the CellQuest Pro software. Parasitemia was determined as the percentage of double stained infected erythrocytes among the whole erythrocyte population. The specific growth inhibitory index (SGI) was calculated according to the following formula: SGI = 100×[1-(percent parasitemia with MN and test IgG/percent parasitemia with test IgG)/(percent parasitemia with MN and naïve IgG/percent parasitemia with naïve IgG)]. An SGI effect was considered as significant if yielding a value >30% [29].

### Protein Structure Prediction

The amino acid sequences of the N-terminal domains of *Pf*SERA1-9 (3D7) were aligned with Multiple Sequence Alignment Tool version 1.1 (http://cib.cf.ocha.ac.jp/KYG/onlyalign.html) [30] after deletion of N-terminal signal sequences predicted by SignalP 3.0 [31]. Since *Pf*SERA8 has no corresponding region, amino acid sequence identity and similarity of relatively conserved regions among *Pf*SERA1-7 and 9 were calculated based on Clustal W alignment and similarity classification in NPS@server (http://npsa-pbil.ibcp.fr/) [32], [33]. Percentage “identity” and “strong similarity” were calculated from the sum of the number of amino acids (Table 1). For prediction of disordered/ordered structure, the sequences were applied to Consensus Disorder Prediction (http://protease.burnham.org/www/tools/html/disorder.html) [34]. Secondary structure prediction of SE36 was performed by Consensus secondary structure prediction in NPS@ server [33].

**Table 1:** Amino acid sequence identity and similarity of the relatively conserved regions (brown bars in Fig. 5) in the N-terminal domain between SE36 (Honduras-1 SERA5) and 3D7 SERA proteins.

3D7 SERA	1	2	3	4	5	6	7	9
Identity (%)	57.2	50.7	54.6	51.3	98.0	53.9	50.7	52.0
Similarity (%)	80.9	77.0	80.9	77.0	99.3	74.3	78.3	80.3

### Circular Dichroism

Circular dichroism (CD) spectra were acquired with a J-820 spectropolarimeter (Jasco, Tokyo, Japan) at 5–37°C. Samples were prepared at 0.2 mg/ml for SE36 or 0.1 mg/ml for peptides in 50 mM sodium phosphate and 150 mM NaCl with or without 40% 2,2,2-trifluoroethanol (TFE). Each spectrum is an average of 20–40 times measurements. The obtained data were converted into mean residue ellipticity, [*θ*]. Peptide concentrations were determined using BCA Protein Assay Kit (Thermo Fisher Scientific). For SE36, the concentration was determined from absorbance at 280 nm using an extinction coefficient calculated as reported by Gill and von Hippel [5], [35].

### Tryptophan Fluorescence

Tryptophan fluorescence spectra were acquired with a F-7000 fluorescence spectrophotometer (Hitachi, Tokyo, Japan) at 25°C with excitation wavelength at 295 nm and emission detection wavelength between 300 to 450 nm. Samples were prepared at 0.2 mg/ml in 50 mM sodium phosphate and 150 mM NaCl with or without 8 M urea.

## Results

### Reactivity of Anti-SE36 Positive Ugandan Serum against the Synthetic Peptides

**Figure 2:**
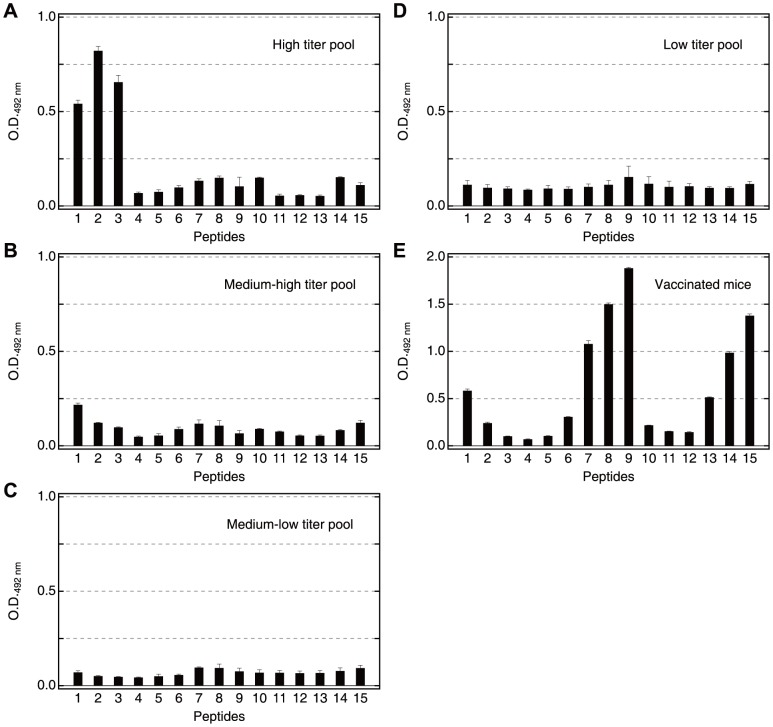
Reactivity of pooled Ugandan serum samples and a vaccinated mouse serum pool against synthetic peptide series I. (**A**) High, (**B**) medium-high, (**C**) medium-low, (**D**) low titer sera pool. Each pool consists of equal aliquots of 9–10 individual sera. The geometric mean anti-SE36 IgG titers of the individual samples are in Table S2. The patterns of reactivity for 18 individual sera are shown in Fig. S2. Serum samples were diluted 800-fold. Secondary antibody was peroxidase-conjugated goat IgG fraction to human IgG (whole molecule) (55220; Cappel ICN Pharmaceuticals Inc, Aurora, OH) diluted 1∶2000. (**E**) Pooled serum from five mice used at 1∶1,600. Secondary antibody was peroxidase conjugated affiniPure goat anti-mouse IgG antibody (H+L) (115-035-166; Jackson ImmunoResearch Laboratories, Inc., West Grove, PA) diluted 1∶5000. All sera were tested for ELISA at least four times. Error bars reflect standard deviation. Reactivity of malaria naïve Japanese serum and naïve mouse serum are shown in Fig. S2.

To determine antigenic regions of SE36, overlapping synthetic peptides corresponding to the N-terminal domain of *Pf*SERA5 (Honduras-1) were prepared (Fig. 1). Based on anti-SE36 IgG levels (Table S2), four pools of high (9 individuals), medium-high (9 individuals), medium-low (9 individuals) and low (10 individuals) titer sera were made and tested for reactivity with peptide series I (Details are in Table S1). As shown in Fig. 2A, pooled high titer sera predominantly reacted with peptides 1, 2 and 3. Peptides 1–3 correspond to the OR and SR regions. Looking at individual sera, more than half of the individuals in the high titer group (or those presumed to have clinical immunity to *P. falciparum* infection) predominantly reacted with peptides 1, 2 and/or 3 (Fig. S2A–D, F, G, I). Medium and low titer pooled sera (Fig. 2B–D), as well as the Japanese naïve control serum (Fig. S2S), reacted poorly to these peptides. In addition, two randomly chosen high titer Ugandan serum samples (PRI and T69) were also examined with another overlapping peptide set (Table S1, series II) which had a different span in the SE36 protein (Fig. S1A, a set of 26 peptides with 20–40 residues). Again, both high titer sera predominantly reacted with peptides 1 and 2, with Ugandan T69 serum also showing reactivity to peptide 3 (Fig. S1B–1 and B–2). This is in contrast to an SE36 vaccinated mouse serum pool that reacted broadly with a number of regions/peptides as shown in Fig. 2E. Reactivity to naïve mouse serum is shown in Fig. S2T.

From Ugandan serum reactivity, it appears that the OR and SR repetitive sequences in the N-terminal regions are highly antigenic in Ugandan adults with high anti-SE36 antibody titers.

### Reactivity of Vaccinated Squirrel Monkey Serum against the Synthetic Peptides

**Figure 3:**
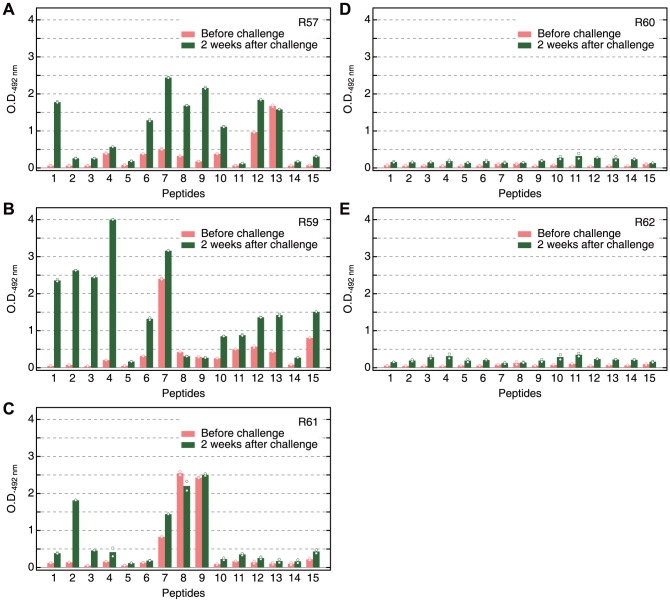
Reactivity in squirrel monkeys. The reactivity of the serum samples from vaccinated (**A–C**) and non-vaccinated (**D, E**) squirrel monkeys against the different peptides in Fig. 1 and Table S1 series I. Red and green bars represent samples before challenge infection and two weeks after challenge infection, respectively. R57, R59-62 are subject codes for squirrel monkeys. Monkey serum were diluted 1∶400; secondary antibody was horseradish peroxidase-conjugated rabbit anti-human IgG antibody (A8792; Sigma-Aldrich Corp., St. Louis, MO) diluted 1∶2000. Mean values from duplicate ELISA with individual data points are shown.

We performed epitope mapping using serum samples obtained previously from SE36 vaccinated squirrel monkeys. Sera from those monkeys vaccinated with SE36 before and after *P. falciparum* challenge infection were used. Before challenge infection, the spectra of reactivity against the peptides were broad, similar to vaccinated mouse serum pool. After challenge infection, some reactivity was observed to the peptides corresponding to OR and SR sequences (Fig. 3A–C). In contrast, monkeys in the control group (without SE36 vaccination) did not show marked response to the synthetic peptides even after challenge infection (Fig. 3D, E). Thus, as earlier reported [5], without SE36 vaccination, no anti-SE36 antibody can be induced despite challenge infection. However, priming the host with SE36 vaccination resulted in a boosting of immune response at the OR and SR sequences by challenge infection. We cannot exclude the presence of other protective epitopes outside the OR and SR sequences (located downstream of the OR and SR sequences) since we did not observe dominant reactivity against OR and/or SR sequences yet all three vaccinated squirrel monkeys were protected from high parasitemia [5].

### Antibody-Dependent Cellular Inhibition (ADCI) Assay with Affinity Purified Antibodies

**Figure 4:**
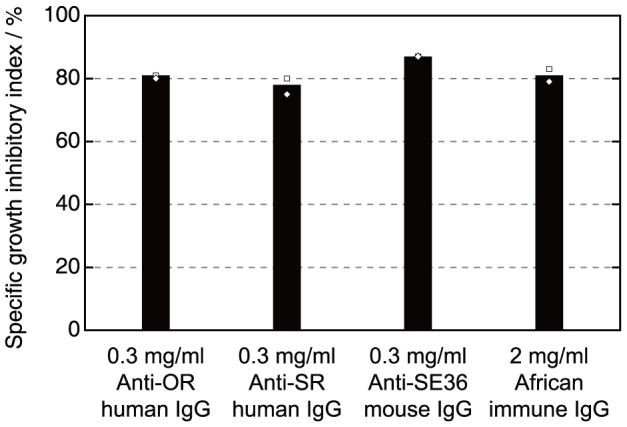
Antibody-dependent cellular inhibition activity with affinity purified antibodies. *In vitro* parasite specific growth inhibition (SGI) in the presence of human monocytes and human affinity-purified anti-OR or anti-SR IgG or murine anti-SE36 IgG at 0.3 mg/ml. A pool of polyclonal immune African IgG from individuals living in endemic areas was used as positive control at a final concentration of 2 mg/ml. IgG purified from malaria naïve human sera was used as negative control and included in the formula for SGI calculation as described in Materials and Methods. Mean values from duplicate ELISA with individual data points are shown.

To examine whether antibodies specific to OR and SR sequences can exert any parasite growth inhibitory effect, we conducted *in vitro* parasite growth inhibition assays with either murine IgG induced by SE36 or affinity purified human natural IgG specific to OR or SR regions in both direct and human monocyte dependent ADCI assays (Fig. 4). The purified human antibodies were tested for their selectivity and reactivity by ELISA (Fig. S1C, D). No significant direct inhibitory effect was observed in all tested IgG. In contrast, anti-parasitic ADCI activity was strong using either induced murine anti-SE36 IgG or human IgG affinity purified against the two synthetic peptides (OR or SR peptide) (Fig. 4). It is noteworthy that these specific IgG preparation used at final concentration of 0.3 mg/ml have a similar growth inhibitory activity to a 2 mg/ml pool of polyclonal immune African IgG previously used in passive transfer experiments of IgG to malaria patients [27], [36]. These results indicate that OR and SR regions are protective epitopes.

### Structure Prediction and Physicochemical Characterization of the N-Terminal Domain of SERA5

**Figure 5:**
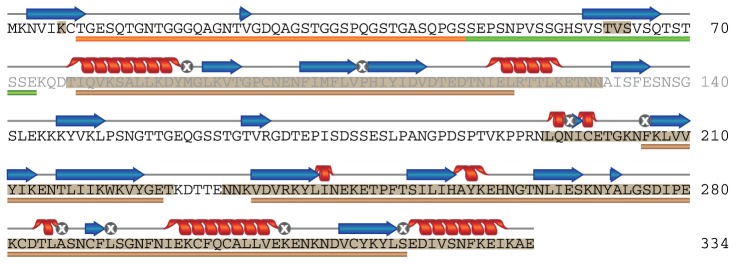
Schematic summary of the sequence analyses of SE36 by structure prediction servers. The shaded amino acids were predicted as ordered residues by the program, Consensus Disorder Prediction. The regions predicted as α-helix and β-structure by NPS@ are represented as helices and arrows above the sequence, respectively. Amino acid denoted by a symbol “⊗”did not reach consensus. The bars in orange, green and brown below the sequence denote OR region, SR region and relatively conserved regions among SERA family genes 1–7 and 9, respectively.

The structural features of both OR and SR sequences as well as the other parts of SE36 protein (based on the SERA5 N-terminal domain of the Honduras-1 strain) were examined using several structure prediction servers. Consensus Disorder Prediction [34] discriminated ordered and disordered regions in the sequence (Fig. 5 and Fig. S3). The region from the N-terminal end to Asp-76 (Fig. 5) was predicted to be predominantly disordered. This region corresponds to OR and SR sequences which were revealed as protective epitopes inducing antibodies capable of parasite growth inhibition. Other disordered regions identified were the polyserine sequence and its N- and C-terminal adjacent regions (matching series I peptides 7–9). All other parts in SE36 were predicted to be ordered. Using the secondary structure prediction program in NPS@ server [33], the assigned secondary structures matched the ordered regions identified with Consensus Disorder Prediction (Fig. 5). The predictions of ordered regions were further confirmed by the fact that all cysteine residues could be aligned at the same positions and high sequence identity and similarity could be obtained in the relatively conserved region shown in Fig. 5 in all of *Pf*SERA family, suggesting a common tertiary structure among the SERA family proteins (Table 1 and Fig. S3).

**Figure 6:**
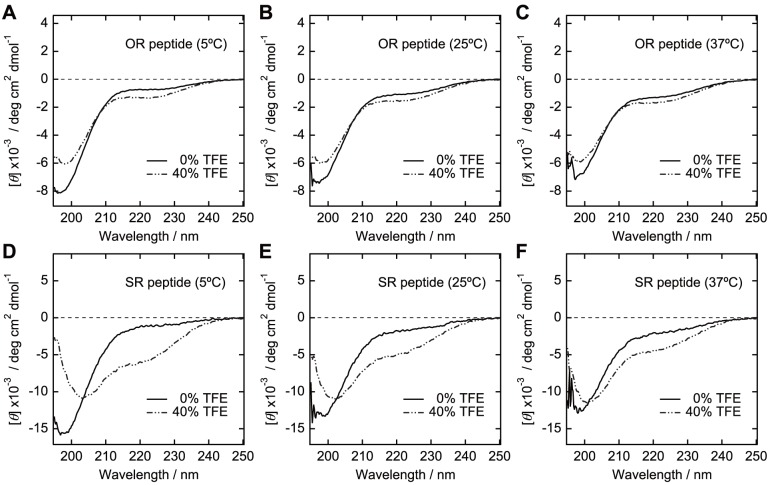
CD spectra of the synthetic peptides. Upper panel (**A–C**) corresponds to OR peptide region. Lower panel (**D–F**) corresponds to SR peptide region. The sequences of the corresponding peptides are in Table S1, series II (peptide 1 and 3, respectively). The measurements were performed at 5, 25 and 37°C with or without 40% TFE.

The synthetic peptides corresponding to OR and SR regions were subjected to CD experiments to define their structural characteristics. The peptide for the OR region did not show any ability to form rigid, typical secondary structure even in the presence of 40% TFE, an inducer and stabilizer for secondary structure (Fig. 6A–C). The behavior of the SR peptide was similar to OR peptide in the absence of TFE. However, with 40% TFE and at lower temperature, the SR peptide showed spectral change distinct from the OR peptide (Fig. 6D–F). Considering the low complexity due to biased amino acid composition of the SR region and the non-significant spectral change, an intrinsically unstructured nature of the region can be suggested. However, the structure predictions also assigned short ordered structure on a cluster of valines (VSTVSVSQ) in the SR region. These results may suggest a possible role of this region for hydrophobic interaction with other molecule(s).

**Figure 7:**
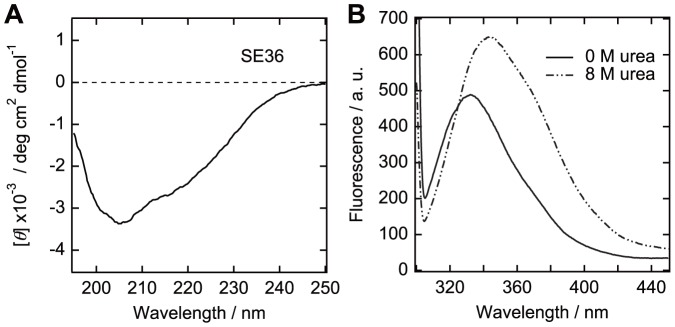
CD (A) and tryptophan fluorescence (B) spectra of SE36. Tryptophan fluorescence measurements were done with or without 8°C.

The CD spectrum of the whole SE36 protein suggests an ordered structure (Fig. 7A). The shoulders near 208 and 222 nm suggest the existence of an α-helical structure. In the above analysis, N-terminal domain of *Pf*SERA5, including SE36, contains only one tryptophan residue that was predicted in an ordered region (Fig. 5). Therefore, tryptophan fluorescence spectra provide a clue to assess the extent of formation of hydrophobic cluster in recombinant SE36 protein. The spectrum of SE36 protein in the absence of denaturant showed a peak at 330–340 nm, suggesting that the region around the tryptophan residue was located in relatively hydrophobic condition (Fig. 7B). The peak of the spectrum shifted to ∼350 nm and the intensity increased upon the addition of 8 M urea. This spectral change implies that fluorescence quenching component(s) such as a polar residue and/or a disulfide bond existed near the tryptophan residue in a non-denatured state. Results from CD and tryptophan fluorescence experiments support that there are some driving forces to form compact tertiary structure in the sequence of SE36. The structure prediction and spectroscopic studies showed the existence of both structured and unstructured parts in SE36 with OR and SR epitopes belonging to unstructured parts.

## Discussion

In this study, we first investigated which sequences in our SE36 vaccine candidate are predominantly recognized by sera from malaria endemic areas. From the results of the epitope mapping using the synthetic peptides, we identified N-terminal repetitive sequences, OR and SR regions, as the dominant epitopes in SE36 protein in Ugandan high titer adult sera. In contrast, vaccination of SE36 to mice or squirrel monkeys did not result in specific induction of OR- and SR-biased antibodies. However, it is interesting that the vaccinated monkeys increased the antibody reactivity against OR and SR regions after challenge infection, while non-vaccinated monkeys did not show any significant response to SE36 even after challenge infection. In malaria endemic areas, as previously reported [5], the frequency of individuals seropositive (or having high antibody titers) to SE36 is not high even in adults. Although the mechanism is unclear, vaccination by SE36 led to induction in immune response to SE36, including OR and SR regions after challenge infection in squirrel monkeys. To examine the inhibitory effect of antibodies against OR and SR dominant epitopes, we performed an ADCI assay. The assay showed that the antibodies specific to both OR and SR regions inhibited in vitro growth of asexual blood stage parasites in cooperation with blood monocytes, suggesting that these regions are indeed protective epitopes in SE36. ADCI has been shown to require for specific inhibition of infected erythrocytes cooperation between the Fc domain of cytophilic IgG and the Fc-γ receptors of monocytes [28], [37]. The recognition and subsequent activation likely induce the release of cytotoxic mediators leading to parasite killing at the intra-erythrocytic level. Although some parasites are indeed phagocytosed [28], this indirect intra-erythrocytic effect is the main mode of action of ADCI. What is generally observed in blood thin smears is the presence of many picnotic or crisis forms of parasites at the end of the assay [37].

The protective epitopes identified in this study are consistent with our previous results using mouse antibodies, i.e., glutathione-S-transferase-fused proteins containing the N-terminal regions corresponding to OR and SR regions were recognized by parasite-inhibitory antibodies [17], [18]. However, the OR and SR regions may not be the sole protective epitopes, since murine SE36 specific antibodies showed broad reactivity against different regions of SE36 and correspondingly have comparable parasite inhibitory effects at similar antibody concentrations (Fig. 2E and Fig. 4). Squirrel monkeys vaccinated with the SE36 protein, likewise, gained measurable protection without showing dominant OR and SR specific antibodies [5]. Additionally, there are also some individual samples (T65, TO28, T64, T68 and TO08) that showed reactivity to other peptide regions (Fig. S2I, L, M, N, P).

We further characterized the physicochemical properties of OR and SR protective epitopes. Structure prediction programs and spectroscopic experiments indicated that the OR and SR regions are predominantly disordered, referred to as IURs. IURs are found in many eukaryotes, especially in apicomplexan parasites including *P. falciparum* [22]. The OR region consists of octamer repeats with closely related sequence motifs and the number of repeats largely differs among 445 field isolates [11]. All of the octamer repeat sequences are similar and lack bulky hydrophobic residues, suggesting that they all are intrinsically unstructured. The sequence motif corresponding to the SR region, on the other hand, is highly conserved although with slight variation in the number of repeats.

One example of the biological function of IURs is interaction of transcription factors with nucleic acids [38], [39]. The fly-casting mechanism has been suggested as an advantage, allowing the flexibility of IURs [40], [41]. Since the OR region has strain-specific octamer repeat numbers [11], the OR region may function to interact with other molecule(s) without strict structural requirement which can be observed in a traditional enzyme-substrate interaction. In contrast to the OR region, the tendency to form a secondary structure at lower temperature with TFE (Fig. 6) and the higher sequence conservation of SR region may reflect rigid structure formation either by binding-coupled folding or more strictly controlled interaction to other molecule(s).

There are a few reports which refer to the immunogenicity of intrinsically unstructured regions [24], [26], [42]. Here, we found strong antigenicity of OR and SR regions of *Pf*SERA5 protein that were found to be intrinsically unstructured. The antibodies recognizing the unstructured peptides have strong antiparasitic effect in an ADCI assay. The intrinsically unstructured characteristic of the protective epitope(s) is an advantage for a vaccine candidate. Some malaria antigens such as PfCP-2.9 (a fusion protein consisting of AMA-1 domain III and AMA-1 19 kDa C-terminal domain fragment) are known to have conformational epitopes which require strict stereo structure of antigens [43]. However, the protective epitopes of SE36 do not require strict stereo structure. Even though the epitopes are unstructured and flexible in free states, upon interacting with an antibody, unstructured peptides would be fixed for antibody specific interactions as shown in Fig. S1C.

## Conclusions

We identified the epitopes targeted by biologically active antibodies in the malaria vaccine candidate SE36 using sera from people living in an endemic area, Uganda. The epitopes have repetitive sequences and have characteristics of intrinsically unstructured region. The polymorphism of the epitope regions is limited and they do not require strict stereo structure to elicit functional antibodies inhibiting *in vitro* growth of asexual blood stage parasites. These results support SE36 as a promising malaria vaccine antigen.

## Supporting Information

Figure S1 **Reactivity studies with peptide series II.**
(DOCX)

Figure S2 **Reactivity studies with peptide series I.**
(DOCX)

Figure S3 **Sequence alignment of** ***Pf*** **SERA1-9.**
(DOCX)

Table S1 **The sequences of synthetic peptides used in this study.**
(DOCX)

Table S2 **Anti-SE36 antibody titers of Ugandan individuals.**
(DOCX)
