# Supplementary material for: Protective Epitopes of the Plasmodium falciparum SERA5 Malaria Vaccine Reside in Intrinsically Unstructured N-Terminal Repetitive Sequences
Source: PLoS One. 2014 Jun 2;9(6):e98460. doi: 10.1371/journal.pone.0098460 (PMC4041889; doi:10.1371/journal.pone.0098460)
Supplement: Figure S1 — Reactivity studies with peptide series II. (DOCX) [file pone.0098460.s001.docx]

**Figure S1. Reactivity studies with peptide series II. (A).** Schematic representation of synthetic peptide series II covering the whole sequence of SE36 protein. Twenty-six peptides, 20-40 residues in length, were chemically synthesized by Toray Research Center (Tokyo, Japan). Peptide 1 and 3, correspond to the OR and SR regions, respectively. Peptide 2 includes overlapping regions of peptide 1 and 3. Peptides 13 and 13’ correspond to a 17-mer dimorphic region. The sequences of peptides 13 and 13’ are found in Honduras-1 strain and K1 strain, respectively [11]. **(B).** Reactivity of Ugandan serum samples (PRI and T69) against the peptide set in panel (A). Serum samples were diluted 1:500; secondary antibody was horseradish peroxidase-conjugated rabbit anti-human IgG antibody (A8792; Sigma-Aldrich Corp., St. Louis, MO) diluted 1:4000. After incubation at room temperature, the plates were washed and color development was performed with TMB Microwell Peroxidase Substrate System (KPL, Inc., Gaithersburg, MD) for 1 minute. The reaction was stopped with 50 µl of 2 M sulfuric acid and optical density was read at 450 nm. **(C).** Recognition specificity of the affinity-purified human IgG used in ADCI assays in concentration dependent ELISA assay. OR peptide corresponds to peptide 1 in panel (A). SR peptide corresponds to peptide 3 in panel (A). Peptide II-13 corresponds to a dimorphic region that is intrinsically unstructured. **(D).** Reactivity of affinity purified anti-OR and anti-SR IgG antibodies against synthetic peptide series I. Affinity purified antibodies were used at concentrations of 0.01 mg/ml; secondary antibody was peroxidase-conjugated goat IgG fraction to human IgG (whole molecule) (55220; Cappel ICN Pharmaceuticals Inc, Aurora, OH) diluted 1:2000. Color development was performed with 100µl freshly prepared citrate-phosphate buffer (pH 5.0) containing 0.2% hydrogen peroxide and OPD tablet (154-01673; Sigma-Aldrich Corp., St. Louis, MO) for 15 minutes. The reaction was stopped with 100 µl of 2 M sulfuric acid and optical density was read at 492 nm. Mean values from duplicate ELISA with individual data points are shown. It was noted that in some experiments, peptide 9 and 10 had absorbance values higher than the average background level. However, the recognition of peptides 9 and 10 by ELISA did not appear as specific or strong as the recognition of peptides 1 to 3.
