# Supplementary material for: Protective Epitopes of the Plasmodium falciparum SERA5 Malaria Vaccine Reside in Intrinsically Unstructured N-Terminal Repetitive Sequences
Source: PLoS One. 2014 Jun 2;9(6):e98460. doi: 10.1371/journal.pone.0098460 (PMC4041889; doi:10.1371/journal.pone.0098460)
Supplement: Figure S2 — Reactivity studies with peptide series I. (DOCX) [file pone.0098460.s002.docx]

**Figure S2. Reactivity studies with peptide series I. (A-R).** Reactivity of 18 representative Ugandan serum samples**.** (A-I) are individual serum samples from the high titer serum pool. (J-R) are individual serum samples from medium-high titer serum pool. **(S).** Malaria naïve Japanese serum. Serum samples were diluted 800-fold. Secondary antibody was peroxidase-conjugated goat IgG fraction to human IgG (whole molecule) (55220; Cappel ICN Pharmaceuticals Inc, Aurora, OH) diluted 1:2000. Mean values from duplicate ELISA with individual data points are shown. Notably, in the high titer group, subjects T49 (E) and T51 (H) were observed to react poorly to peptide series I. We speculate that for these two sera the antibodies recognizing conformational epitopes contributed to give high antibody titer readings against whole SE36 molecule. **(T).** Reactivity of naïve mouse serum. Mouse serum was used at 1:1,600. Secondary antibody was peroxidase conjugated affiniPure goat anti-mouse IgG antibody (H+L) (115-035-166; Jackson ImmunoResearch Laboratories, Inc., West Grove, PA) diluted 1:5000. Mean values from duplicate ELISA with individual data points are shown.
