# Supplementary material for: Protective Epitopes of the Plasmodium falciparum SERA5 Malaria Vaccine Reside in Intrinsically Unstructured N-Terminal Repetitive Sequences
Source: PLoS One. 2014 Jun 2;9(6):e98460. doi: 10.1371/journal.pone.0098460 (PMC4041889; doi:10.1371/journal.pone.0098460)

**Figure S3. Sequence alignment of *Pf*SERA1-9 (sequences of the 3D7 strain are derived from PlasmoDB).** The order of the SERA family members follow sequence similarity relative to SERA5 as determined from the output of Multiple Sequence Alignment Tool [30]. Amino acids are classified and colored by their properties. “⚫” denotes completely conserved amino acid, “⭘” denotes highly conserved amino acid with one or two exceptions, “+” denotes conserved amino acid property, and a red rectangle denotes conserved cysteine residues. *Pf*SERA8 was excluded from the judgment of degrees of conservation because its N-terminal domain is extraordinarily short compared with those of the other *Pf*SERA proteins. The shaded amino acids are those predicted as ordered residues. “↓” shows the C-terminal end of *Pf*SERA5 and SE36.


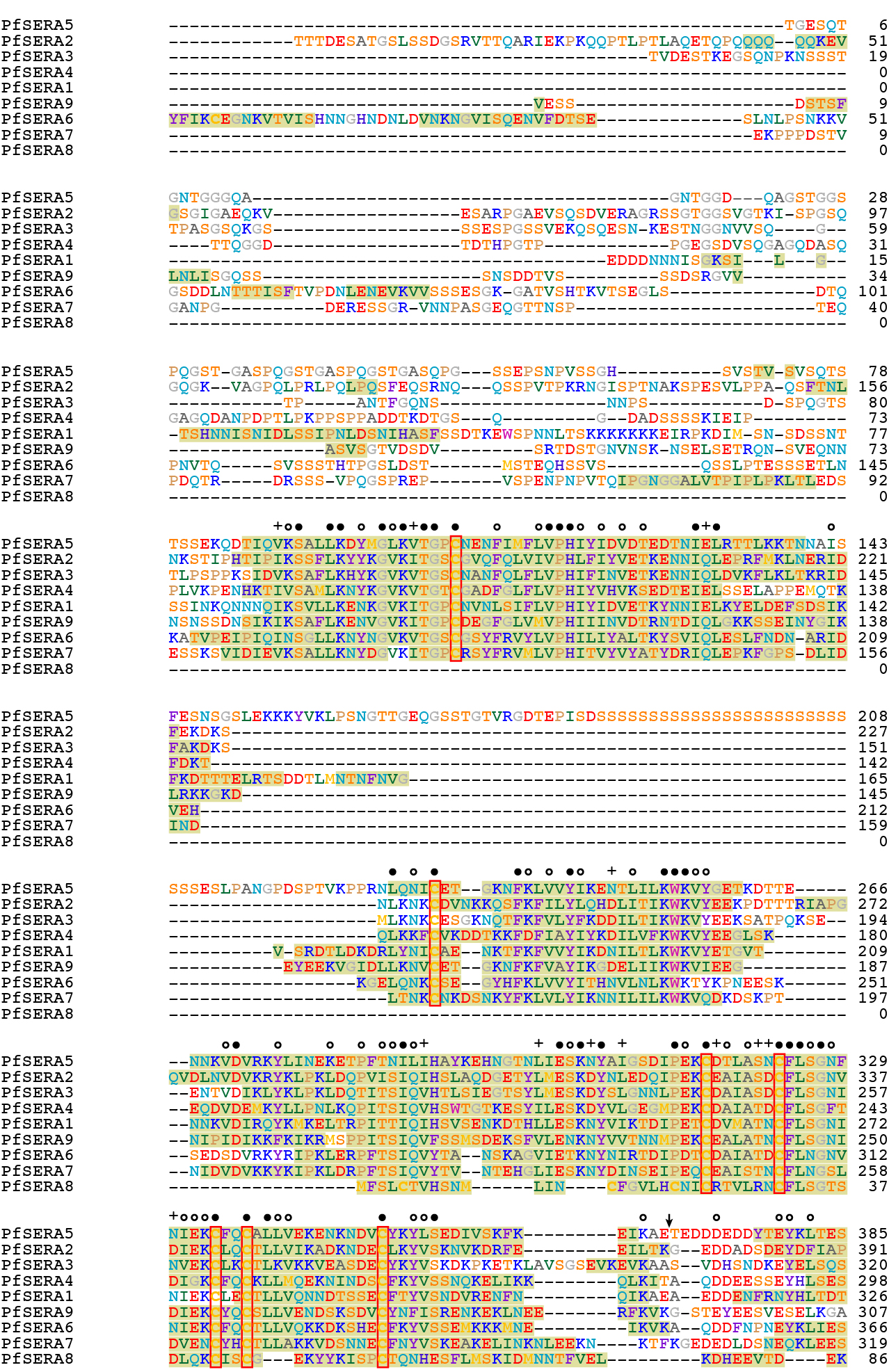

Supplement: Figure S3 — Sequence alignment of Pf SERA1-9. (DOCX) [file pone.0098460.s003.docx]
