# Supplementary material for: Protective Epitopes of the Plasmodium falciparum SERA5 Malaria Vaccine Reside in Intrinsically Unstructured N-Terminal Repetitive Sequences
Source: PLoS One. 2014 Jun 2;9(6):e98460. doi: 10.1371/journal.pone.0098460 (PMC4041889; doi:10.1371/journal.pone.0098460)
Supplement: Table S1 — The sequences of synthetic peptides used in this study. (DOCX) [file pone.0098460.s004.docx]

**Table S1. The sequences of synthetic peptides used in this study.**

| Series I | Sequence |
| --- | --- |
| Peptide 1 | MKNVIKCTGESQTGNTGGGQAGNTVGDQAGSTGGSPQGSTGA |
| Peptide 2 | NTVGDQAGSTGGSPQGSTGASQPGSSEPSNPVSSGHSVSTVS |
| Peptide 3 | SQPGSSEPSNPVSSGHSVSTVSVSQTSTSSEKQDTIQVKSAL |
| Peptide 4 | VSQTSTSSEKQDTIQVKSALLKDYMGLKVTGPCNENFIMFLV |
| Peptide 5 | LKDYMGLKVTGPCNENFIMFLVPHIYIDVDTEDTNIELRTTL |
| Peptide 6 | PHIYIDVDTEDTNIELRTTLKETNNAISFESNSGSLEKKKYV |
| Peptide 7 | KETNNAISFESNSGSLEKKKYVKLPSNGTTGEQGSSTGTVRG |
| Peptide 8 | KLPSNGTTGEQGSSTGTVRGDTEPISDSSESLPANGPDSPTV |
| Peptide 9 | DTEPISDSSESLPANGPDSPTVKPPRNLQNICETGKNFKLVV |
| Peptide 10 | KPPRNLQNICETGKNFKLVVYIKENTLIIKWKVYGETKDTTE |
| Peptide 11 | YIKENTLIIKWKVYGETKDTTENNKVDVRKYLINEKETPFTS |
| Peptide 12 | NNKVDVRKYLINEKETPFTSILIHAYKEHNGTNLIESKNYAL |
| Peptide 13 | ILIHAYKEHNGTNLIESKNYALGSDIPEKCDTLASNCFLSGN |
| Peptide 14 | GSDIPEKCDTLASNCFLSGNFNIEKCFQCALLVEKENKNDVC |
| Peptide 15 | FNIEKCFQCALLVEKENKNDVCYKYLSEDIVSNFKEIKAE |

| Series II | Sequence |
| --- | --- |
| Peptide 1 | CTGESQTGNTGGGQAGNTVGDQAGSTGGSPQGSTGASQPGS |
| Peptide 2 | CTGGSPQGSTGASQPGSSSEPSNPVSSEPSNPVSSGHSVS |
| Peptide 3 | CSSEPSNPVSSGHSVSTVSVSQTSTSSEKQ |
| Peptide 4 | CTSTSSEKQDTIQVKSALLK |
| Peptide 5 | CQVKSALLKDYMGLKVTGPC |
| Peptide 6 | CGLKVTGPCNENFIMFLVPH |
| Peptide 7 | CFIMFLVPHIYIDVDTEDTN |
| Peptide 8 | CDVDTEDTNIELRTTLKETN |
| Peptide 9 | CRTTLKETNNAISFESNSGS |
| Peptide 10 | CSFESNSGSLEKKKYVKLPSN |
| Peptide 11 | CKYVKLPSNGTTGEQGSSTGT |
| Peptide 12 | CEQGSSTGTVRGDTEPISDSS |
| Peptide 13 | CSESLPANGPDSPTVKPPRN |
| Peptide 13’ | CSVNPPANGAGSTPDAKKKN |
| Peptide 14 | CPTVKPPRNLQNICETGKNFK |
| Peptide 15 | CETGKNFKLVVYIKENTLII |
| Peptide 16 | CIKENTLIIKWKVYGETKDT |
| Peptide 17 | CVYGETKDTTENNKVDVRKY |
| Peptide 18 | CNKVDVRKYLINEKETPFTS |
| Peptide 19 | CEKETPFTSILIHAYKEHNG |
| Peptide 20 | CHAYKEHNGTNLIESKNYAL |
| Peptide 21 | CIESKNYALGSDIPEKCDTLA |
| Peptide 22 | CPEKCDTLASNCFLSGNFNIE |
| Peptide 23 | CLSGNFNIEKCFQCALLVEKE |
| Peptide 24 | CALLVEKENKNDVCYKYLSE |
| Peptide 25 | CVCYKYLSEDIVSNFKEIKAE |
