# Supplementary material for: Protective Epitopes of the Plasmodium falciparum SERA5 Malaria Vaccine Reside in Intrinsically Unstructured N-Terminal Repetitive Sequences
Source: PLoS One. 2014 Jun 2;9(6):e98460. doi: 10.1371/journal.pone.0098460 (PMC4041889; doi:10.1371/journal.pone.0098460)
Supplement: Table S2 — Anti-SE36 antibody titers of Ugandan individuals. (DOCX) [file pone.0098460.s005.docx]

**Table S2. Anti-SE36 antibody titers of Ugandan individuals.***

| Group | Rank | Subject No. | Geometric mean | Exp. 1 | Exp. 2 |
| --- | --- | --- | --- | --- | --- |
| High | 1 | T41 | 13725.4 | 15471.8 | 12176.1 |
|  | 2 | PRI | 8292.4 | 8148.5 | 8438.9 |
|  | 3 | T69 | 8040.2 | 8173.3 | 7909.4 |
|  | 4 | T54 | 3936.0 | 4222.7 | 3668.8 |
|  | 5 | T49 | 3334.0 | 3412.1 | 3257.7 |
|  | 6 | T44 | 2785.3 | 2961.6 | 2619.4 |
|  | 7 | TO03 | 2582.2 | 2565.9 | 2598.6 |
|  | 8 | T51 | 2460.9 | 2426.2 | 2496.1 |
|  | 9 | T65 | 2296.7 | 2321.7 | 2272.0 |
| Medium-  high | 10 | TO21 | 2265.4 | 2322.1 | 2210.1 |
|  | 11 | TO18 | 2235.4 | 2299.1 | 2173.5 |
|  | 12 | TO28 | 2206.9 | 2275.0 | 2140.8 |
|  | 13 | T64 | 1858.5 | 1903.5 | 1814.5 |
|  | 14 | T68 | 1412.5 | 1400.0 | 1425.0 |
|  | 15 | TO05 | 1301.5 | 1293.5 | 1309.5 |
|  | 16 | TO08 | 1247.9 | 1295.2 | 1202.4 |
|  | 17 | TO12 | 1162.5 | 1150.9 | 1174.2 |
|  | 18 | T40 | 1162.0 | 1268.4 | 1064.6 |
| Medium-  low | 19 | T60 | 707.5 | 711.8 | 703.2 |
|  | 20 | T37 | 640.8 | 675.5 | 607.8 |
|  | 21 | TO04 | 473.8 | 462.3 | 485.6 |
|  | 22 | T36 | 471.2 | 496.9 | 446.7 |
|  | 23 | T63 | 331.7 | 318.5 | 345.3 |
|  | 24 | TO11 | 274.1 | 277.1 | 271.2 |
|  | 25 | T59 | 257.4 | 250.5 | 264.6 |
|  | 26 | TO26 | 191.1 | 195.6 | 186.6 |
|  | 27 | T47 | 190.2 | 199.4 | 181.4 |
| Low | 28 | T67 | 187.2 | 180.8 | 193.8 |
|  | 29 | T70 | 182.4 | 186.8 | 178.1 |
|  | 30 | TO07 | 101.0 | 103.0 | 99.1 |
|  | 31 | T34 | 83.7 | 99.8 | 70.2 |
|  | 32 | T55 | 64.3 | 58.0 | 71.1 |
|  | 33 | T35 | 63.7 | 75.2 | 53.9 |
|  | 34 | TO01 | 27.3 | 23.3 | 31.9 |
|  | 35 | TO29 | 22.7 | 20.4 | 25.3 |
|  | 36 | TO23 | 22.7 | 23.7 | 21.7 |
|  | 37 | T58 | 7.9 | 6.0 | 10.5 |

* Antibody titers are relative to a standard high titer serum pool that was included on each ELISA plate in order to generate a standard curve. A 5000 unit value was assigned as the reciprocal of the dilution giving an O.D._492 nm_ = 1 in a standardized assay. Based on the antibody titer of the standard serum, the titer of each serum was calculated with an equilibrium line assay (Bioassay Assist software ver. 2.0.7).
